# Sleep analysis results of portable polysomnography in patients with acute and chronic temporomandibular disorder

**DOI:** 10.26502/droh.0052

**Published:** 2022-10-07

**Authors:** Yeon-Hee Lee, Q-Schick Auh

**Affiliations:** Department of Orofacial Pain and Oral Medicine, Kyung Hee University Dental Hospital, Kyung Hee Medical center, Kyung Hee University, Seoul, Korea

**Keywords:** Biopsychosocial, Chronic, Depression, Polysomnography, Sleep, Temporomandibular disorder

## Abstract

**Objective:**

This study aimed to investigate portable polysomnography (PSG)-based ‘sleep’ and pre-diagnosis of obstructive sleep apnoea (OSA) in acute temporomandibular disorder (TMD) and patients with chronic TMD.

**Methods:**

Randomly selected 25 patients with acute TMD (mean age, 42.58 ± 18.77 years; 14 females) and 26 age-and sex-matched patients with chronic TMD (mean age, 49.24 ± 17.52 years, 19 females) were enrolled.

**Results:**

The eight psychological subscales of SCL-90R had significantly higher values in the chronic TMD group than in the acute TMD group (all p < 0.05). There was no significant group difference in the respiratory event index examined using a portable PSG. OSA was observed in 57.7% in acute TMD, and 68.0% in chronic TMD, respectively. From the multiple regression analysis, palpation index was the strongest predictor of pre-diagnosis of OSA (OR = 17.550). Among the contributing factors for TMD, psychological stress (OR = 12.226), self-reported sleep problems (OR = 10.222), and above-average value of DEP (OR = 1.443) were followed.

**Conclusion:**

Patients with chronic TMD were psychologically more vulnerable than those with acute TMD, and the existence of subjectively perceived sleep problems or objective sleep indices examined by portable PSG could affect TMD symptom severity in different ways.

## Introduction

Temporomandibular disorder (TMD) is a broad term characterised by pain and dysfunction in the temporomandibular joint (TMJ), masticatory muscles, and surrounding structures. Among the general population, 60–70% have at least one sign of TMD, 12% have TMD-related pain, and 5% seek treatment [1]. TMD occurs 1.5–2 times more often in females than in males, with 80% of patients treated for TMD are females [2]. The aetiology of TMD is multifactorial, with the two main factors being physical and psychological. Therefore, the world’s most widely used diagnostic criteria for TMD (DC/TMD) involve the assessment of two axes: physical factors (Axis I) and possible psychological factors (Axis II) [3]. Typical and frequent symptoms of TMD include TMJ noise and TMD-related pain, followed by restricted mandibular movement, headache, neck pain, ear pain, and tinnitus. Although the aetiology of TMD is multifactorial, it shifts from a mechanically based phenomenon to a biopsychosocial model of chronic TMD pain [4]. Plausible risk factors for TMD include recurrent microtrauma, macrotrauma, other body pain conditions, psychological status, sleep problems, and genetic factors [5].

The mechanism of the transition from acute to chronic TMD is unclear. However, the underlying mechanisms related to the clinical features of patients with acute TMD and chronic TMD may be different [6,7]. As pain becomes chronic, psychological aspects become more vulnerable and sleep problems accompany it, which is also the case in TMD [8]. Unresolved psychological disorders (e.g., depression and anxiety) can cause muscle tension that can lead to clenching and grinding of teeth, which can lead to TMD [9]. Acute TMD pain occurs suddenly and usually responds to traditional management and treatment. Pain can persist for a long time beyond what the patient can cope with, and underlying biological and psychological processes may contribute [10]. Therefore, chronic TMD can present persistent, recurrent, or chronic pain associated with TMJ and/or muscles involved in the masticatory system [11]. Although access to chronic TMD is challenging, the clinician must prevent the chronicity of TMD signs and symptoms through comparative studies of patients with acute and chronic TMD.

Studies are still lacking on whether obstructive sleep apnoea (OSA), a classic sleep-disordered respiration, is closely related to the onset and chronicity of TMD. OSA is characterised by intermittent obstruction of the upper airways during sleep and recurrent apnoea and hypopnea, leading to intermittent hypoxia and sleep fragmentation and impaired sleep quality [12]. OSA is very common in adults, with one in five adults having at least mild OSA and one in 15 adults having moderate or more OSA [13]. OSA-related symptoms include daytime sleepiness, decreased cognitive ability, decreased quality of life, increased risk of accidents, and cardiovascular side effects, which cause social and personal burdens. In a previous prospective cohort study, a high probability of OSA was associated with a higher incidence of first-onset and chronic TMD [14]. OSA causes the airway to collapse, causing the body to push the lower jaw forward and lift the airway, and this constant mandibular movement overnight can cause stress and strain on the TMJ and adjacent muscles. However, the downside of this study is that OSA risk was measured using a sleep questionnaire rather than an objective sleep evaluation. Although there have been expert opinions on the relationship between TMD, orofacial pain, and OSA [15], objective, evidence-based studies remain weak.

The gold standard diagnostic route for patients with suspected sleep apnoea is sleep laboratory referral for overnight polysomnography (PSG). The PSG procedure involves continuous recording of various physiological data, including airflow, chest/abdominal movement, electroencephalography, electro-ophthalmoscopy, electromyography, electrocardiography, and oxyhemoglobin saturation [16]. Apnoea and hypopnea were detected with the monitoring equipment during the sleep period, and the number of events per hour was summed to generate an apnoea–hypopnea index (AHI). However, PSG is not readily available in sleep laboratories in many areas because it is expensive, cumbersome, and difficult to perform repeatedly. Evaluating a patient’s sleep and OSA with a portable monitoring device based on a limited number of channels in a home-like environment may reduce costs and improve access to treatment. Self-administered patient testing may be the preferred method for obtaining the necessary physiological data. The American Sleep Disorders Association (ASDA) classified the diagnostic system into four categories according to the test environment, technician presence, and number of parameters recorded [17]. ASDA level III, for use in the present study, was reserved for devices that monitor airflow, chest/abdominal movement, heart rate/pulse rate, and oxyhemoglobin saturation.

To date, research in this field has focused mainly on chronic TMD. However, to better understand the predispositions that may contribute to the transition to chronic TMD, factors that influence the symptoms of acute TMD must also be investigated. Therefore, we investigated and compared the demographics, clinical characteristics, psychological factors, and contributing factors of TMD in patients with acute and chronic TMD. We also investigated sleep and pre-diagnosis of OSA using a level III portable PSG. We hypothesized that the clinical characteristics of acute and chronic TMD may be different and that psychological and sleep factors will be more impaired in patients with chronic TMD. Finally, we examined factors associated with the severity of TMD symptoms.

## Materials and Methods

### Participants

The research protocol was reviewed in accordance with the Helsinki Declaration and approved by the Institutional Review Board of Kyung Hee University Dental Hospital (KHD IRB no.1804–2). Written informed consent was obtained from all participants. Informed consent was obtained to publish de-identified images of one participant who performed portable PSG in an online open–access publication.

To investigate the research purpose, the authors designed and conducted a randomised controlled study. We identified patients with TMD who visited the Department of Orofacial Pain and Oral Medicine at Kyung Hee University Dental Hospital of Seoul from 1 June 2018 to 31 March 2022. The patients were diagnosed with TMD using DC/TMD for TMD Axis I [3]. When pain persists for >3–6 months, individuals are usually considered in a chronic state [18]. We assigned patients who experienced TMD signs and symptoms lasting <6 months for ‘acute TMD’ and those after >6 months of onset as ‘chronic TMD.’ Among the 83 patients with TMD with sleep reports, 25 patients with acute TMD and 26 age-and sex-matched patients with chronic TMD were randomly selected. We retrospectively reviewed all sleep and TMJ reports and patients with any level of data loss were excluded from the study.

The study sample consisted of patients with TMD according to the inclusion and exclusion criteria. The inclusion criteria for patients with TMD were as follows: completed a set of routine TMJ assessments, patients who performed portable PSG and completed sleep reports, as well as constructive questionnaires, and no treatment of the current episode other than medication. The exclusion criteria were as follows: serious injuries, such as facial fracture and unstable multiple traumas, previous injury, neurological disorder not related to the trauma, musculoskeletal disorder before the injury, rheumatism, psychological problems, and pregnancy. To assess the impact of TMD chronicity on the distribution of demographics, clinical factors for signs and symptoms of TMD, psychological factors, and presence of OSA, all variables were compared by group.

### TMD classification and clinical evaluation

Clinical evaluation procedures included an oral examination, interview, panoramic radiography, and a comprehensive questionnaire in DC/TMD Axis I diagnostic algorithms for the diagnosis of TMD. Diagnostic algorithms for myofascial pain, disc displacement, arthralgia, and headache attributed to TMD are based on DC/TMD Axis I diagnostic algorithms.

### Characteristics of pain

Patients reported the duration of symptoms in the masticatory muscles, TMJ, and adjacent structures regarding the number of days elapsed since the patient first experienced symptoms related to TMD. TMD-related pain was scored subjectively by the patients, ranging from 0 (no pain at all) to 10 (the worst pain imaginable) using the visual analogue scale (VAS).

### Palpation index (PI), neck PI, and dysfunction index (DI)

PI is a reliable scoring system that analyses the severity of myofascial pain, allowing an objective evaluation of TMD symptoms. In each patient, we palpated 20 intraoral and extraoral muscle sites and three sites in the neck. The index-finger palpation pressure was 1 kg/cm^2^ and was applied for 5 s. To calibrate the palpation pressures, we regularly pretested our index-finger pressure using a hand-held pressure algometer. A binary answer (yes/no) was provided for each site. To calculate the PI score, we added all positive responses and divided the sum by the number of events, as described in a previous study [5]. To further investigate the intensity of neck pain, we calculated the neck PI, which was defined as the number of positive responses to the palpation of the neck muscles, including the sternocleidomastoid, splenius capitis, and trapezius muscles, divided by the number of events. Using these two indices, we quantified clinical myofascial pain. DI was used to quantify TMD-related mandibular dysfunction and pain in the TMJ region. DI was defined as the number of positive answers regarding mandibular movements, joint noise, and joint capsule sensitivity divided by the number of events.

### Contributing factors for TMD

We investigated self-reported parafunctional activities using the Oral Behaviour Checklist, including bruxism [19]. Self-assessment of sleep problems, headaches, psychological stress, and tinnitus has also been reported. Self-reported macrotrauma experience was evaluated using the dichotomous question, ‘Do you have any macrotrauma experience associated with current TMD?’ Each parameter was recorded as a binary answer (yes or no) for all patients.

### Psychological evaluation with SCL-90R

Psychological characteristics were evaluated using the Symptom Checklist-90-Revised (SCL-90R) scale. SCL-90R comprises nine symptom subscales: somatisation (SOM), obsessive-compulsiveness (O-C), interpersonal sensitivity (I-S), depression (DEP), anxiety (ANX), hostility (HOS), phobic anxiety (PHOB), paranoid ideation (PAR), and psychosis (PSY), as well as three global indices of functioning, including the global severity index (GSI), positive symptom distress index (PSDI), and positive symptom total (PST).

### Portable PSG index and pre-diagnosis of OSA

**Figure 1:**
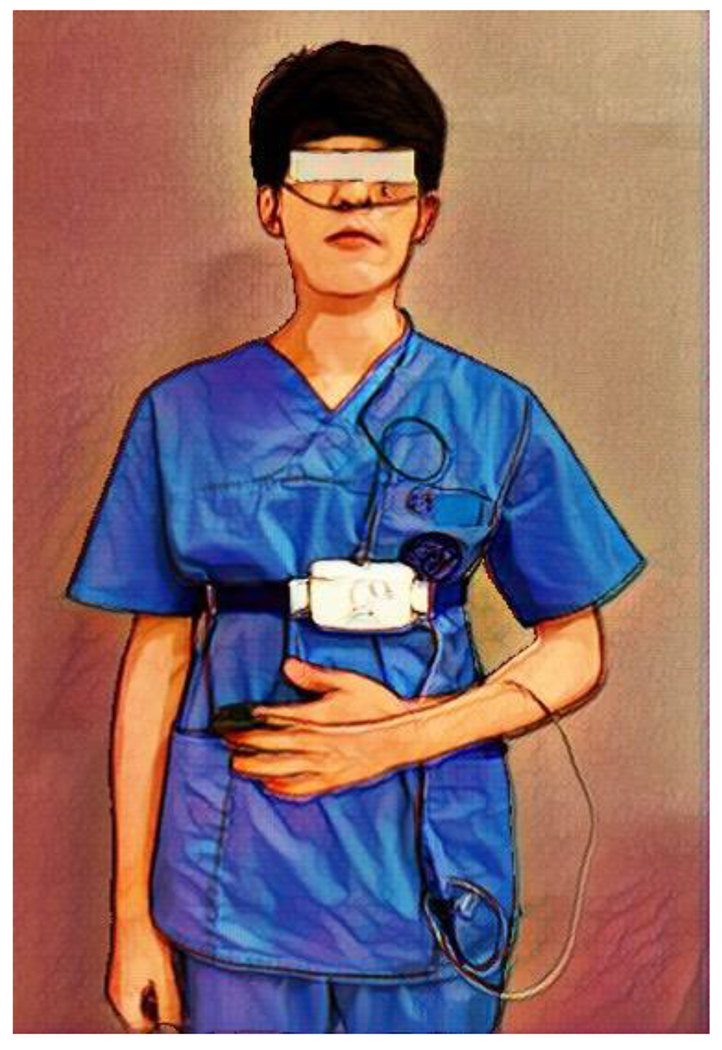
Portable PSG including nasal cannula, thoracic and abdominal respiratory effort, body position sensor, and pulse oximetry

We identified and diagnosed OSA using The Alice OneNight (Philips, Amsterdam, The Netherlands). Alice OneNight home sleep testing is a portable level 3 polysomnography device used to test sleep apnoea at home. It includes oxygen saturation (SpO_2_, finger probe, and oximetry board Nonin), pulse rate (from oximeter probe), airflow (pressure-based airflow with detection of snore through a nasal cannula and thermistor), thoracic and abdominal effort (inductance plethysmography), and body position (Figure 1). The patient installed and operated this device on his or her body for more than one day, and the operator selected representative data for one day without data loss and used it for analysis.

To pre-diagnose OSA, the respiratory event index (REI) was used. The REI represents the number of apnoeas and hypopneas detected by the portable monitoring device per hour of elapsed recording time. The central apnoea index (CAI), obstructive apnoea index (OAI), and mixed apnoea index (MAI) were added, and each index calculated the number of events per hour. Apnoea was defined as a 90% reduction in airflow for at least 10 s, and hypopnea was defined as a ≥30% reduction in airflow for at least 10 s, associated with a ≥3% reduction in oxygen saturation. OSA was defined as an REI ≥5 /hour and classified as mild (REI 5.0–14.9 /hour), moderate (REI 15.0–29.9 /hour), severe (REI ≥30 /hour), and normal (REI <5 /hour) [20]. Sleep studies were considered acceptable for data analysis if none of the following rejection criteria occurred: (1) a portable monitoring device elapsed time less than 2 h or (2) poor quality PSG recording (defined as a substantial portion of the PSG not interpretable for the scoring of sleep and respiratory events).

### Data analysis

The descriptive statistics presented percentages, means and standard deviations (SDs) for continuous variables. Student’s t-test for nonnormally distributed variables was used to compare the acute and chronic TMD groups. Differences in the means of continuous variables between the independent groups were examined using the Student’s t-test. The chi-square test and Fisher’s exact test with Bonferroni correction were used to determine the equality of proportions. The chi-square test and Pearson’s correlation test were used to analyze bivariate correlations between categorical and continuous variables. Kappa statistics were used to measure the degree of agreement (Kappa coefficient) between the two examiners who evaluated and rated the same subjects. Multiple regression analysis was performed with OSA as a dependent variable and the presence of demographics, clinical factors, psychological factors, and contributing factors for TMD as an independent variable. We also investigated the increased risk of OSA in patients with chronic versus acute TMD. Odds ratios (ORs) with 95% confidence intervals (CIs) and p-values were investigated. Spearman’s correlation analysis was performed to investigate the relationship between TMD indices and other variables. Statistical significance was set at a two-tailed p-value < 0.05. Data were analysed using IBM SPSS Statistics for Windows (version 20.0; IBM Corp., Armonk, NY, USA).

## Results

### Demographics and TMD index

The average age of all patients with TMD was 45.84 ± 18.29 years old, and the female (n = 33) to male (n = 18) ratio was 1.83:1, with a high female ratio. Table 1 compares the demographics and TMD indices between the acute and chronic TMD groups. There was no significant difference in age difference and male and female composition of the acute TMD and chronic TMD groups with age-and sex-matched composition. The mean TMD symptom duration was significantly longer in the chronic TMD group than in the acute TMD group (40.77 ± 48.85 days vs. 1261.60 ± 1590.11 days, p < 0.001).

Regarding the TMD index for the quantification of TMD symptoms, VAS, neck PI, and DI did not differ significantly. In the case of PI, the incidence of chronic TMD was significantly higher than that of acute TMD (0.147 ± 0.094 vs. 0.253 ± 0.107, p < 0.001).

### Contributing factors for TMD

Among the contributing factors to TMD that we investigated, there was no factor with a difference in distribution between the two groups. When the presence of each factor was investigated, headache was the most frequent contributing factor (n = 28, 54.9%), psychological stress (n = 27, 52.9%), sleep problem (n = 17, 33.3%), bruxism (n = 11, 21.6%), tinnitus (n = 10, 19.6%), and macrotrauma history (n = 9, 17.6%) were followed.

### Psychological factors using SCL-90R

Among the nine SCL-90R subscales, the mean value of eight items, excluding PAR, was significantly higher in chronic TMD than in acute TMD. That is, the mean value of including SOM (46.50 ± 5.13 vs. 53.72 ± 10.45, p < 0.01); O-C (43.04 ± 6.64 vs. 53.32 ± 7.82, p < 0.001); I-S (45.65 ± 7.28 vs. 54.40 ± 10.07, p < 0.001); DEP (44.19 ± 7.34 vs. 54.68 ± 9.56, p < 0.001); ANX (46.15 ± 7.66 vs. 55.04 ± 10.19, p < 0.01); HOS (44.92 ± 5.30 vs. 52.48 ± 9.11, p < 0.01); PHOB (44.85 ± 2.87 vs. 52.88 ± 15.04, p < 0.05); PSY (44.23 ± 3.66 vs. 52.20 ± 8.71, p < 0.001) was significantly higher than in chronic TMD compared to acute TMD. All three composite indicators, GSI, PDSI, and PST, were significantly higher in chronic TMD than in acute TMD (Table 2).

### Results with portable PSG

**Figure 2:**
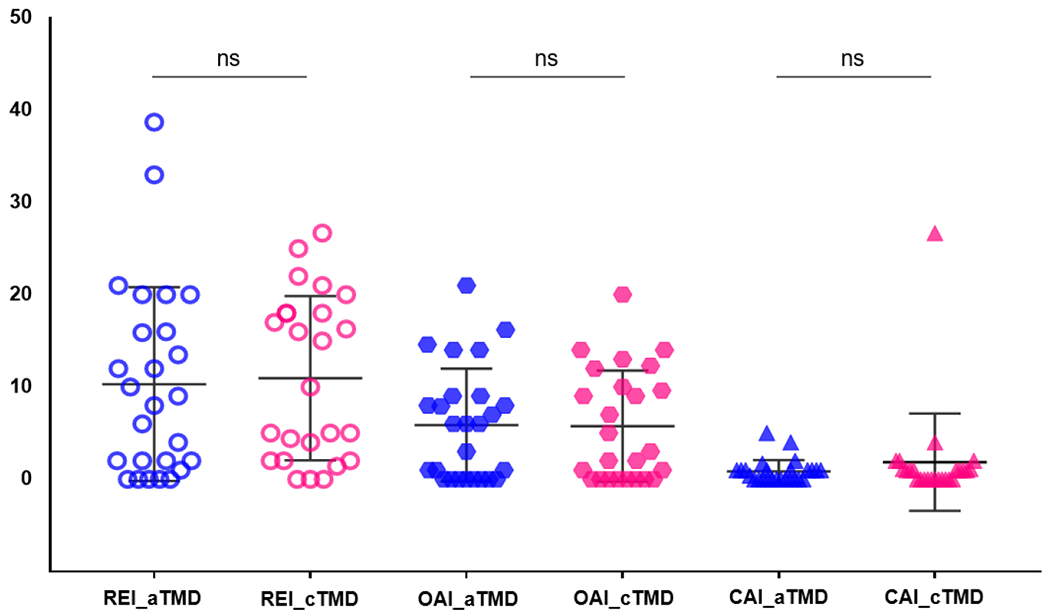
Comparison between TMD groups in portable PSG index aTMD, acute TMD group; cTMD, chronic TMD group; REI, respiratory event index; OAI, obstructive apnoea index; CAI, central apnoea index. The results were obtained using a t-test. Statistical significance was set at P < 0.05. A p-value ≥ 0.05 indicated by “ns” for not significant.

Table 3 shows the objective sleep and pre-diagnosis of OSA using portable PSG. Regarding the portable PSG index, the mean values of OAI, CAI, MAI, hypopnea, and REI were not significantly different between the two groups (Figure 2). For all patients with TMD, the mean value of REI was 10.63 ± 9.65. The REI values of acute TMD were 10.31 ± 10.50, and that of chronic TMD was 10.95 ± 8.89, respectively. The lowest SpO_2_ values were not significantly different between the acute and chronic TMD groups (86.35 ± 4.66 vs. 84.04 ± 14.13, p = 0.404).

A pre-diagnosis of OSA was observed in 32 patients with TMD (72.7%). OSA was diagnosed in 68.0% of the patients in the chronic TMD group, which was higher than that of the acute TMD group (57.7%), but the difference was not statistically significant (p = 0.605). In both the acute and chronic TMD groups, moderate OSA (30.8% and 44.0%, respectively) was observed with greater frequency than mild OSA (26.9% and 24.0%, respectively). Severe OSA was not observed in any case of the two TMD groups.

### Regression analysis for predicting OSA

Multiple regression analysis was performed to analyse predictive factors, and the pre-diagnosis of OSA was assumed as a dependent variable (Table 4). The pre-diagnosis of OSA was confirmed based on the REI score (≥5) obtained from the portable PSG test. The previously investigated demographics, TMD index, psychological factors, and contributing factors for TMD were included as independent variables. From the results of the multiple regression analysis, PI (above-average value) was the strongest predictor of pre-diagnosis of OSA (OR = 17.550). Among the contributing factors for TMD, psychological stress (OR = 12.226), self-reported sleep problems (OR = 10.222), and above-average value of DEP (OR = 1.443) were followed.

### Correlations among the TMD indices

Significant positive correlations were observed between neck PI, PI, and DI in both the acute TMD and chronic TMD groups. In the acute TMD group, neck PI and PI had a strong positive correlation (r = 0.566, p < 0.01), and PI and DI also had a positive correlation (r = 0.441, p < 0.05). Similarly, positive correlations were observed between neck PI and PI (r = 0.623, p < 0.01) and between PI and DI (r = 0.513, p < 0.01) in the chronic TMD group. In both the acute and chronic TMD groups, the higher the PI, the higher the neck PI and DI. In both groups, there was no correlation between neck PI, which indicates the degree of myofascial pain in the neck, and DI, which indicates TMJ dysfunction and pain (Table 5).

### Correlation between clinical characteristics and the TMD index

In the acute TMD group, bruxism was positively correlated with the DI score (r = 0.418, p < 0.05). In the chronic TMD group, bruxism (r = 0.540, p < 0.01) and self-reported sleep problems (r = 0.471, p <0.05) were positively correlated with the VAS score. Interestingly, psychological factors examined with SCL-90R showed a correlation with PI and neck PI. In the acute TMD group, DEP (r = 0.406), ANX (r = 0.436), and PSY (r=0.414) were positively correlated with an increase in PI. In chronic TMD, the factors correlated with neck PI were SOM (r = 0.449, p < 0.05), ANX (r = 0.414, p < 0.05), PHOB (r = 0.421, p < 0.05), and PAR (r=0.434, p <0.05). The factors positively correlated with PI were SOM (r = 0.623, p < 0.01), O-C (r = 0.476, p < 0.05), I-S (r = 0.590, p < 0.01), DEP (r = 0.566, p < 0.01), ANX (r = 0.571, p < 0.01), and PHOB (r = 0.730, p < 0.01). The nine psychological subscales of SCL-90R were not significantly correlated with VAS and DI. Thus, it is inferred that muscular pain and psychological factors are more closely related than joint pain (Table 5).

### Correlation between the portable PSG index and the TMD index

A significant correlation was observed in the portable PSG index in the acute TMD group. In the acute TMD group, the lowest SpO_2_ was significantly negatively correlated with PI (r = −0.525, p < 0.01). CAI was positively correlated with PI (r = 0.553, p < 0.01) and neck PI (r = 0.459, p < 0.05). Hypopnea was positively correlated with REI (r = 0.511, p < 0.01) and DI (r = 0.454, p < 0.05) (Table 5).

## Discussion

Psychological aspects, the existence of subjectively perceived sleep problems, or objective sleep indices examined by portable PSG could affect TMD symptom severity in different ways in acute and chronic TMD. In our study, PI was significantly higher in patients with chronic TMD than in those with acute TMD. Interestingly, patients with chronic TMD were psychologically more vulnerable than those with acute TMD. The eight psychological subscales of SCL-90R, except for PAR, including SOM, O-C, I-S, DEP, ANX, HOS, PHOB, and PSY, had significantly higher values in chronic TMD than in acute TMD. Furthermore, OSA was observed in many patients with TMD (n = 32, 62.7%). In the multiple regression analysis, the ‘TMD group’ was not a significant predictor of OSA. PI was the strongest predictor of OSA (OR = 17.550), followed by psychological stress (OR = 12.226), self-reported sleep problems (OR = 10.222), and DEP (OR = 1.443). In acute TMD, the decrease in the lowest SpO_2_ value and an increase in CAI were correlated with an increase in the PI score. In chronic TMD, an increase in REI was correlated with an increase in DI.

The severity of temporomandibular myofascial pain, measured by PI, was significantly higher in the chronic TMD group than in the acute TMD group. Conversely, DI, which quantifies TMJ pain and dysfunction, did not differ between the acute and chronic TMD groups. As TMD pain becomes chronic, the effect of pain of myogenous origin rather than arthrogenous origin may increase. TMD pain of myofascial origin is difficult to diagnose and treat and is considered a chronic pain disorder [21]. The overall prevalence of myofascial TMD pain is up to 45.3%, and TMD itself is also commonly considered a chronic pain condition [22]. General treatment of temporomandibular myofascial pain in our clinical environment combines pharmacological therapy, stabilisation splint therapy, cognitive therapy, and meditation, which produces relief of symptoms. However, no consensus protocol for the treatment of temporomandibular myofascial pain has been established. In our previous study, myofascial pain was associated with poor sleep quality in patients with chronic TMD [8]. More approaches to sleep and biopsychosocial aspects are needed to manage TMD pain of myogenous origin.

The pathophysiological mechanism of TMD has shifted from a mechanistic-based theory to a biopsychosocial model, particularly in patients with chronic TMD [23,24]. In the present study, the distribution of TMD contributing factors, such as bruxism and history of macrotrauma, did not differ significantly between acute and chronic TMD. According to the International Classification of Sleep Disorders, the classification criteria for sleep bruxism include the objective signs and symptoms observed by clinicians [25]. However, this study was conducted based on self-reported bruxism and its interpretation was limited. Unsolved psychological problems such as anxiety and depression can cause an increase in muscle tension that can lead to tooth clenching or bruxism, which in turn can lead to the onset and persistence of TMD [9]. In addition, macrotrauma, such as whiplash injury, may be associated with the chronicity of TMD pain because a series of peripheral and central sensitisation and psychological damage from macrotrauma occur [26,27]. In this study, a macrotrauma history did not increase the risk of OSA and did not significantly affect the symptom severity of TMD.

OSA is a common sleep disorder that leads to hypoxemia and sleep fragmentation. Interrupted breathing attributed to OSA leads to a reduced level of oxygen in the blood, which can trigger inflammation [28]. OSA has been associated with many medical conditions, such as cardiovascular diseases, juvenile idiopathic arthritis, and neuromuscular disorders [29,30]. OSA patients with chronic pain also have higher pain levels, higher disability levels, and lower quality of life [31]. OSA more than tripled the incidence of chronic TMD in a previous OPPERA study [32]. Sleep disturbances due to hypoxia and sleep fragmentation reliably predict new occurrences and exacerbations of chronic pain [33]. In this study, the prevalence of OSA in patients with TMD was 62.7%, and it occurred more frequently in patients with chronic TMD (68.0%) than in those with acute TMD (57.7%). Regarding the occurrence of OSA in patients with TMD, abnormal movement of the lower jaw can increase the myofascial pain intensity and tightness of the surrounding muscles [34]. Only in chronic TMD was the increase in REI correlated with an increase in DI. Poor sleep quality is very common in patients with TMD and has been observed in up to 90% of patients [35]. OSA is a common comorbidity in the general population with chronic pain, with an overall prevalence of approximately 37%, and is reported to be approximately 29% in patients with TMD [36]. Poor sleep quality was strongly associated with chronic TMD pain [8]. However, the relationship between OSA and TMD chronicity was unclear in this study.

Furthermore, the PI score was the strongest predictor of OSA (OR = 17.550) in patients with TMD, followed by psychological stress (OR = 12.226), self-reported sleep problems (OR = 10.222), and DEP (OR = 1.443). Myofascial pain in TMD is associated with a mild increase in sleep fragmentation [37]. In acute TMD, the decrease in the lowest SpO_2_ value and an increase in CAI were correlated with an increase in the PI score. Repeated intermittent hypoxia or ischaemia ultimately leads to muscle damage [38]. Decreased sleep quality, pain, and psychological vulnerability interact inseparably with each other. Compared with healthy controls, patients with OSA had more perceived psychological stress, and stress was closely related to anxiety and depressive symptoms [39]. Furthermore, psychological stress contributes to the formation of trigger points for pain-causing muscle tension and myofascial pain [40]. Although somewhat complicated, clinicians should always consider the effects of nocturnal hypoxemia and fragmented sleep during OSA on pain intensity in patients with TMD inpatient screening. Furthermore, based on the biopsychosocial model, factors such as psychological stress, depression, and myofascial pain should be considered in patients with chronic pain. More studies are needed to determine how each of the various factors affects acute and chronic TMD and how they have complex correlations.

This study had several limitations. Although the portable SPG device has a simpler configuration than the general PSG, patients still feel uncomfortable even when they fall asleep with the device installed on their body in a comfortable environment at home. This discomfort caused a decrease in the total sleep time and monitoring time. Additionally, since this study was conducted on patients who agreed to perform portable PSG among those who visited during the study period, bias may have occurred in selecting patients with TMD.

## Conclusion

When comparing the acute and chronic TMD groups, the most striking difference was that psychological parameters were more impaired in the chronic TMD group than in the acute TMD group. Psychological factors, such as SOM, O-C, I-S, DEP, ANX, and PHOB, increase myofascial pain intensity in patients with chronic TMD and negatively affect them. There was no direct difference between the TMD groups in the portable PSG index, and there was no significant difference in OSA incidence and REI scores. However, in the acute TMD group, a decrease in the lowest SpO_2_ and an increase in CAI were associated with an increase in myofascial pain intensity, while an increase in REI and CAI was associated with an increase in TMJ pain and dysfunction. Therefore, when screening patients with TMD, clinicians must consider that the biopsychosocial profile may differ depending on signs and symptoms, and the underlying mechanisms or related body systems may be different.

## Figures and Tables

**Table 1:** Epidemiology and clinical characteristics of patients with TMD

	Acute TMD (n = 26)	Chronic TMD (n = 25)	p-value
	mean ± SD or n (%)	mean ± SD or n (%)	
Demographics			
Age (years)^a^	42.58 ± 18.77	49.24 ± 17.52	0.197
Sex^b^			
Female	14 (53.8%)	19 (76.0%)	0.086
Male	12 (46.2%)	6 (24.0%)	
**Symptom duration (days)** ^a^	**40.77 ± 48.85**	**1261.60 ± 1590.11**	**<0.001** ^***^
TMD index			
VAS (0-10) ^a^	5.73 ± 2.01	5.52 ± 2.14	0.719
Neck PI (0-1) ^a^	0.331 ± 0.211	0.404 ± 0.226	0.238
**PI (0-1)** ^a^	**0.147 ± 0.094**	**0.253 ± 0.107**	**<0.001** ^***^
DI (0-1) ^a^	0.459 ± 0.245	0.523 ± 0.208	0.316

TMD, temporomandibular disorder; VAS, visual analogue scale; PI, palpation index; DI, dysfunction index; SD, standard deviation.

Statistical significance was set at p < 0.05;

***: p < 0.001

a: The results were obtained using t-test;

b: The results are obtained using the chi-square test.

**Table 2:** Distribution of contributing factors and psychological profiles

	Acute TMD (n = 26)	Chronic TMD (n = 25)	p-value
	mean ± SD or n (%)	mean ± SD or n (%)	
*Contributing factor*			
Bruxism ^a^	5 (19.2%)	6 (24.0%)	0.743
Self-reported sleep problem ^a^	10 (38.5%)	7 (28.0%)	0.555
Headache ^a^	15 (57.7%)	13 (52.0%)	0.781
Psychological distress ^a^	14 (53.8%)	13 (52.0%)	1
Tinnitus ^a^	6 (23.1%)	4 (16.0%)	0.726
Macrotrauma ^b^	3 (11.5%)	6 (24.0%)	0.291
*SCL-90R*			
**SOM** ^c^	**46.50 ± 5.13**	**53.72 ± 10.45**	**0.003** ^**^
**O-C** ^c^	**43.04 ± 6.64**	**53.32 ± 7.82**	**<0.001** ^***^
**I-S** ^c^	**45.65 ± 7.28**	**54.40 ± 10.07**	**<0.001** ^***^
**DEP** ^c^	**44.19 ± 7.34**	**54.68 ± 9.56**	**<0.001** ^***^
**ANX** ^c^	**46.15 ± 7.66**	**55.04 ± 10.19**	**0.001** ^**^
**HOS** ^c^	**44.92 ± 5.30**	**52.48 ± 9.11**	**0.001** ^**^
**PHOB** ^c^	**44.85 ± 2.87**	**52.88 ± 15.04**	**0.010** ^*^
PAR ^c^	44.69 ± 4.84	47.44 ± 8.56	0.162
**PSY** ^c^	**44.23 ± 3.66**	**52.20 ± 8.71**	**<0.001** ^***^
**GSI** ^c^	**43.35 ± 5.80**	**54.16 ± 9.70**	**<0.001** ^***^
**PDSI** ^c^	**46.54 ± 5.78**	**50.32 ± 8.25**	**0.005** ^**^
**PST** ^c^	**42.23 ± 9.25**	**57.01 ± 7.03**	**<0.001** ^***^

TMD, temporomandibular disorder; SOM, somatisation; O-C, obsessive-compulsiveness; I-S, interpersonal sensitivity; DEP, depression; ANX, anxiety; HOS, hostility; PHOB, phobic anxiety; PAR, paranoid ideation; PSY, psychosis; GSI, global severity index; PSDI, positive symptom distress index; PST, positive symptom total.

Statistical significance was set at p < 0.05;

*: p < 0.05;

**: p < 0.01;

***: p < 0.001

a: The results are obtained using the chi-square test;

b: The results are obtained using Fisher’s exact test and Bonferroni correction;

c: The results are obtained using t-test.

**Table 3:** Objective sleep and pre-diagnosis of OSA investigation with portable PSG

	Acute TMD (n = 26)	Chronic TMD (n = 25)	p-value
	mean ± SD or n (%)	mean ± SD or n (%)	
Total recording time (min) ^a^	265.86 ± 182.56	281.24 ± 183.30	0.765
Portable PSG index			
Lowest SpO_2_ (%) ^a^	86.35 ± 4.66	84.04 ± 14.13	0.404
OAI (events/hour) ^a^	5.87 ± 6.11	5.76 ± 6.04	0.945
CAI (events/hour) ^a^	0.85 ± 1.24	1.84 ± 5.27	0.358
MAI (events/hour) ^a^	0.62 ± 0.89	0.72 ± 0.75	0.64
Hypopnea (events/hour) ^a^	3.09 ± 4.62	2.52 ± 2.64	0.594
REI (events/hour) ^a^	10.31 ± 10.50	10.95 ± 8.89	0.815
OSA severity			
Normal (REI <5) ^b^	11 (42.3%)	8 (32.0%)	0.605
OSA (REI ≥5)	15 (57.7%)	17 (68.0%)	
1) Mild OSA (REI 5.0–14.9)	7 (26.9%)	6 (24.0%)	
2) Moderate OSA (REI 15.0–29.9)	8 (30.8%)	11 (44.0%)	

TMD, temporomandibular disorder; PSG, polysomnography; SpO2, oxygen saturation; OAI, obstructive apnoea index; CAI, central apnoea index; MAI, mixed apnoea index; REI, respiratory event index; OSA, obstructive sleep apnoea

a: The results are obtained using t-test;

b: The results are obtained using the chi-square test. Statistical significance was set at p < 0.05.

**Table 4:** Multiple regression analysis to predict OSA in patients with TMD

Pre-diagnosis of OSA in patients with TMD (n = 51)				
	OR	95% CI lower	95% CI upper	p-value
***Demographics***				
Age [ref.=under average value]	1.293	0.158	10.565	0.81
Female [ref.=Male]	0.469	0.061	3.622	0.468
Chronic TMD [ref.=Acute TMD]	1.425	0.187	10.868	0.733
***TMD index***				
VAS	0.794	0.255	0.476	0.691
Neck PI [ref.=under average value]	7.185	0.623	82.888	0.114
**PI [ref.=under average value]**	**17.55**	**0.582**	**528.929**	**0.009** ^**^
DI [ref.=under average value]	0.449	0.081	2.505	0.361
***SCL-90R***				
SOM [ref.=under average value]	0.964	0.774	1.201	0.743
OC [ref.=under average value]	0.889	0.726	1.088	0.254
IS [ref.=under average value]	1.114	0.899	1.381	0.323
**DEP [ref.=under average value]**	**1.443**	**0.989**	**2.106**	**0.048** ^*^
ANX [ref.=under average value]	0.831	0.641	1.079	0.164
HOS [ref.=under average value]	0.646	0.453	0.921	0.056
PHOB [ref.=under average value]	0.991	0.825	1.19	0.924
PAR [ref.=under average value]	0.79	0.573	1.09	0.151
PSY [ref.=under average value]	1.204	0.919	1.579	0.178
***Contributing factors***				
Bruxism [ref.=none]	0.649	0.092	4.564	0.664
**Self-reported sleep problem [ref.=none]**	**10.222**	**1.481**	**70.564**	**0.018** ^*^
Headache [ref.=none]	0.354	0.068	1.856	0.219
**Psychological distress [ref.=none]**	**12.226**	**1.277**	**117.084**	**0.017** ^*^
Tinnitus [ref.=none]	0.528	0.066	4.209	0.547
Macrotrauma [ref.=none]	0.547	0.065	4.618	0.579

OSA, obstructive sleep apnoea; OR, odds ratio; CI, confidence interval; TMD, temporomandibular disorder; VAS, visual analogue scale; PI, palpation index; DI, dysfunction index; SOM, somatisation; O-C, obsessive-compulsiveness; I-S, interpersonal sensitivity; DEP, depression; ANX, anxiety; HOS, hostility; PHOB, phobic anxiety; PAR, paranoid ideation; PSY, psychosis

The results were obtained using multiple regression analysis. Statistical significance was set at p < 0.05;

*: p < 0.05;

**: p < 0.01

**Table 5:** Factors that correlate with TMD symptom severity

Spearman’s r		Acute TMD (n = 26)				Chronic TMD (n = 25)			
		VAS	Neck PI	PI	DI	VAS	Neck PI	PI	DI
**TMD index**	**Neck PI**	−0.081		**0.566** ^**^	0.031	−0.206		**0.623** ^**^	0.299
	**PI**	0	**0.566** ^**^		**0.441** ^*^	0.045	**0.623** ^**^		**0.513** ^**^
	**DI**	−0.078	0.031	**0.441** ^*^		0.045	0.299	**0.513** ^**^	
**Contributing factor**	**Bruxism**	0.293	0.055	−0.114	**0.418** ^*^	**0.540** ^**^	0.257	0.309	0.121
	**Sleep problem**	0.158	0.089	−0.013	−0.197	**0.471** ^*^	−0.014	0.014	0.194
	**Headache**	0.078	−0.031	0.065	−0.055	0.199	0.045	0.045	0.045
	**Stress**	0.309	0.187	0.283	0.012	0.359	0.368	0.116	0.116
	**Tinnitus**	0	0.177	0.228	0.27	0.017	0.167	0.053	0.053
	**Macrotrauma**	0.12	0.263	0.241	0.309	0.022	0.121	0.257	0.257
**SCL-90R**	**SOM**	0.109	0.207	0.062	0.037	0.329	**0.449** ^*^	**0.623** ^**^	0.219
	**O-C**	0.216	0.276	0.34	0.141	0.184	0.409	**0.476** ^*^	0.14
	**I-S**	0.047	0.196	0.292	0.183	0.1	0.354	**0.590** ^**^	0.23
	**DEP**	0.211	0.27	**0.406** ^*^	0.146	0.25	0.347	**0.566** ^**^	0.073
	**ANX**	0.263	0.341	**0.436** ^*^	0.23	0.05	**0.414** ^*^	**0.571** ^**^	0.073
	**HOS**	0.338	0.082	0.384	0.29	0.184	0.191	0.37	0.034
	**PHOB**	0.054	0.062	0.064	0.103	0.223	**0.421** ^*^	**0.730** ^**^	0.247
	**PAR**	0.199	−0.011	0.21	−0.065	0.142	**0.434** ^*^	0.326	0.051
	**PSY**	0.282	0.33	**0.414** ^*^	0.058	0.028	0.281	0.298	0.101
**Portable PSG index**	**Lowest SpO_2_**	0.026	−0.271	−0.525^**^	−0.125	0.206	−0.017	−0.129	−0.062
	**OAI**	0.036	−0.268	−0.068	0.317	0.023	−0.148	0.176	0.119
	**CAI**	−0.216	0.076	**0.533** ^**^	**0.459** ^*^	0.212	−0.101	0.296	0.053
	**MAI**	−0.022	−0.098	0.268	0.279	−0.179	−0.18	0.041	−0.174
	**Hypopnea**	−0.026	−0.148	0.102	**0.511** ^**^	−0.135	0.04	0.141	0.017
	**REI**	−0.046	−0.228	0.078	**0.454** ^*^	−0.089	0.022	0.336	0.146

TMD, temporomandibular disorder; VAS, visual analogue scale; PI, palpation index; DI, dysfunction index; SOM, somatisation; O-C, obsessive-compulsiveness; I-S, interpersonal sensitivity; DEP, depression; ANX, anxiety; HOS, hostility; PHOB, phobic anxiety; PAR, paranoid ideation; PSY, psychosis; PSG, polysomnography; SpO_2_, oxygen saturation; OAI, obstructive apnoea index; CAI, central apnoea index; MAI, mixed apnoea index; REI, respiratory event index.

The results are obtained using Spearman’s correlation analysis. Spearman’s r indicates the correlation between two factors (Spearman’s r: −1 to 1). The larger the absolute value, the stronger the correlation. In this table, red indicates a positive correlation, and green indicates a negative correlation. Statistical significance was set at p < 0.05;

*: p < 0.05;

**: p < 0.01

## Data Availability

Data supporting the findings of this study are available upon request from the corresponding author [Y.-H.L.].
